# P077 Carotidynia and large vessel vasculitis: an unusual presentation and rare complication of granulocyte-colony stimulating factor therapy

**DOI:** 10.1093/rap/rkag113.107

**Published:** 2026-11-03

**Authors:** Bhagya Arun, Abdelhameed Yousif, Roopa Prasad

**Affiliations:** Rheumatology Department, University Hospital of Wales, Cardiff, United Kingdom; Rheumatology Department, University Hospital of Wales, Cardiff, United Kingdom; Rheumatology Department, University Hospital of Wales, Cardiff, United Kingdom

## Abstract

**Introduction:**

Granulocyte-colony stimulating factor (GCSF) is often used in haematology settings for the management of neutropaenia. GCSF-associated large vessel vasculitis (LVV) is a rare complication. We report a patient with chronic lymphocytic leukaemia (CLL) who developed acute neck pain within days of initiating GCSF. CT and subsequent PET-CT imaging demonstrated widespread large vessel vasculitis, including involvement of both carotid arteries. GCSF treatment was discontinued and he responded to glucocorticoids, with resolution of the changes seen on imaging. This case highlights the importance of awareness of GCSF-associated LVV and recognition of unusual presenting features, to avoid diagnostic delay or misdiagnosis.

**Case description:**

A 68-year-old man with CLL, diagnosed in 2010, under haematology follow-up for 14 years, at high infection risk due to previous disseminated aspergillosis and severe COVID-19 pneumonitis.

Surveillance CTTAP in June 2024 showed progressive splenomegaly, enlarging iliac lymphadenopathy, normal aorta. In September 2024, he developed worsening fatigue and had rising WBC (115 ×10⁹/L) and lymphocyte count (>100 ×10⁹/L). He was started on reduced dose venetoclax due to his infection risk.

He was admitted 2 weeks later with sore throat and generally unwell. His CRP was normal but he had progressive neutropaenia. GCSF injections (filgrastim) and broad spectrum antibiotics were commenced; venetoclax was discontinued after four weeks. GCSF was administered on alternate days for one week and then daily.

Within 12 days of starting GCSF, he developed left-sided neck pain and tender left submandibular swelling. CT scan demonstrated new mural thickening along both carotid arteries and left subclavian artery, suggestive of inflammation. He had no other features suggestive of systemic vasculitis. GCSF was discontinued due to suspicion of GCSF-induced LVV. CRP was now 199 mg/L; infection screen and vasculitis screen, including ANCA and autoimmune antibodies, were negative.

He received one pulse of 80mg IV methylprednisolone while awaiting PET-CT scan. This demonstrated FDG-avid inflammation in the left subclavian artery, aortic arch, abdominal aorta and both femoral arteries. There was no FDG-avid nodal disease and his splenomegaly had improved.

Prednisolone 60 mg daily was initiated; within days, his carotidynia resolved and CRP normalized. At 4 weeks review, he remained well with normal CRP. Prednisolone was tapered and discontinued after 3 months. Repeat PET-CT, done one month after steroid discontinuation, showed complete resolution of the previous findings. He remains asymptomatic off glucocorticoids; CT neck and CT TAP in April 2026 showed no recurrence of LVV, no splenomegaly or lymphadenopathy.

**Discussion:**

GCSF is widely used in haematology to prevent chemotherapy-related neutropenia. Commonly used agent are filgrastim (short-acting, daily injection) and pegfilgrastim (long-acting, PEG-conjugated form). LVV has emerged as an uncommon but increasingly recognised adverse effect of GCSF treatment since the first report by Darie et al. in 2004.

GCSF–associated LVV typically presents with fever, neck or chest pain and raised inflammatory markers. Symptoms usually occur within days of exposure, with an average onset of 5 days (range 1–8) after the last dose. Imaging demonstrates circumferential arterial wall thickening on ultrasound, CT, or MRI and FDG uptake on PET-CT.

The underlying mechanism remains uncertain; proposed pathways include cytokine-driven neutrophil activation and endothelial injury. The prolonged cytokine exposure in patients receiving high doses or long-acting GCSF, is a predisposing factor.

Our case illustrates the diagnostic complexity in an immunocompromised patient with CLL, neutropaenia, high background infection risk and recent GCSF exposure. Our patient’s predominant symptoms were of carotidynia, secondary to the carotid artery inflammation seen on CT scan. Further imaging with PET-CT scan demonstrated the LVV was more widespread, in a patient was otherwise systemically well. Differential diagnoses included idiopathic LVV, infection, and malignancy. The patient’s age, absence of cranial symptoms, negative infection screen and PET-CT findings argued against these alternatives. The temporal association, with early onset of carotidynia following initiation of GCSF and rapid improvement on drug withdrawal and glucocorticoid treatment, strongly supported GCSF–associated LVV.

Management involves discontinuing GCSF and initiating glucocorticoids. Awareness of the diagnosis led to prompt discontinuation of GCSF, initiation of glucocorticoids and facilitated more rapid tapering of prednisolone in a patient with high infection risk.

As in our patient, most cases resolve clinically and radiologically within months. Prognosis is generally favourable, though aortic dissection and aneurysm are reported complications.

**Key learning points:**

• Drug induced LVV is an important and under-recognised complication in patients receiving GCSF for haematological malignancies.

• Clinical presentation may be non-specific. Our patient’s presenting symptom was neck pain, due to carotidynia, secondary to vasculitis affecting the carotid arteries.

In immunocompromised patients, fatigue and fever with raised inflammatory markers may be misattributed to infection or disease progression, triggering infection work-up and delaying recognition of vasculitis.

• Awareness of GCSF-associated LVV is crucial in both haematology and rheumatology settings as prompt investigation is central to diagnosis.

CT or MRI may show mural thickening of the aorta, carotid or subclavian arteries. PET-CT is highly sensitive, demonstrating FDG-avid inflammation, differentiating vasculitis from infection or malignancy.

• The temporal association with GCSF exposure is a key diagnostic clue. Prompt discontinuation of GCSF therapy is essential. Glucocorticoids remain the mainstay of treatment, with most patients responding rapidly. Slow tapering is recommended, balancing relapse risk against infection risk in immunocompromised individuals.

• PET-CT is valuable for monitoring treatment response, often showing complete resolution within months.

• Multidisciplinary management is crucial to avoid unnecessary antimicrobial treatment or inappropriate escalation of immunosuppression.

• Our patient had an excellent outcome with prompt recognition and treatment, with full clinical and radiological resolution. There has been no recurrence at 12 months following glucocorticoid cessation.

This case contributes to the currently limited literature on GCSF-associated LVV in several ways. Our patient had unusual presenting symptoms with carotidynia; he developed LVV within days of starting GCSF; he had received shorting acting GCSF; he was relatively asymptomatic though PET-CT scan indicated widespread large vessel involvement, including both carotid arteries, left subclavian artery, the thoracic and abdominal aorta and both femoral arteries.

